# Helping alveolar macrophages live to fight another day during viral pneumonia

**DOI:** 10.1172/JCI201457

**Published:** 2026-01-16

**Authors:** Elise M.R. Armstrong, Joseph P. Mizgerd

**Affiliations:** 1 Pulmonary Center,; 2 Department of Virology, Immunology, and Microbiology,; 3 Department of Medicine, and; 4 Department of Biochemistry and Cell Biology, Boston University Chobanian and Avedisian School of Medicine, Boston, Massachusetts, USA.

## Abstract

Alveolar macrophages (AMs) help defend the lungs against infection, but during pneumonia many alveolar macrophages die. In this issue of the *JCI*, Malainou et al. explored the mechanism underpinning AM death during viral pneumonia and its effect on the outcomes of bacterial superinfection, a secondary infection that occurs before the first infection is cleared. In mouse models of influenza A infection, recruited neutrophils secreted TNF superfamily member 14 (TNFSF14), and AMs increased expression of the TNFSF14 receptors TNFSFR14 and type I transmembrane lymphotoxin β receptor (LTβR). TNFSF14 signaling via the LTβR was sufficient to cause AM apoptosis. TNFSF14 deficiency or blockade preserved AMs during influenza infection and diminished bacterial burdens and mouse mortality during pneumococcal superinfection. The adoptive transfer of AMs decreased the severity of pneumococcal superinfections, if those AMs lacked the LTβR. Thus, preserving AMs by interrupting TNFRSF14-LTβR interactions can make virus-infected lungs less susceptible to severe bacterial superinfection.

## Alveolar macrophages and pneumonia

Alveolar macrophages (AMs) are constituent cells of the lung that are essential to health and homeostasis (1, 2). Humans or mice lacking AMs due to interrupted granulocyte macrophage–colony-stimulating factor (GM-CSF) signaling, which can occur as a result of genetic deficiencies or autoantibodies, have pulmonary alveolar proteinosis and are susceptible to lower respiratory infections, implicating AM roles in host defense. The defensive role of AMs is borne out by experiments in which the targeted deletion of AMs makes respiratory infections more severe. AMs have many roles that together help protect the lungs during infection. They patrol the alveoli to sound the alarm upon recognizing pathogens, they ingest and kill microbes, and they enhance the resolution and repair of injured lungs via efferocytosis of dying cells and nurturing of the epithelium.

The pool of AMs in the lungs is dynamic. In early life, AMs are products of the embryonic hematopoietic system and are long lived and capable of self-renewal. With age, growing fractions of the AM pool are instead derived from postnatal monocyte precursors. Severe lung infections rapidly accelerate this process by causing widespread death of AMs as well as by promoting the recruitment of monocytes, which differentiate into macrophages (from which a portion survive past the infection as AMs). The AMs in lungs that recover from infection differ from the AMs of naive lungs in many ways, with altered surface markers, metabolomes, transcriptomes, and responses to subsequent infections (3–7). The resting states and responses of AMs are highly influenced by prior exposures.

Respiratory viral infections cause a transient susceptibility to bacterial superinfection. Because AMs have essential roles in host defense against bacterial pneumonia, including phagocytosis, sentry, resolution, and repair activities, Malainou et al. considered the possibility that the transient loss of AMs during viral pneumonia may contribute to bacterial superinfection severity (8). They endeavored to define mechanisms underlying the AM loss, manipulate the pathways responsible, and test for functional significance.

## TNF superfamily member TNFSF14 signaling mediates AM death

After infecting mice with influenza A virus, Malainou et al. (8) observed that the peak loss of AM correlated temporally with susceptibility to pneumococcal infection. AMs in the influenza-infected mouse lung increased programmed cell death–related transcripts, as well as caspase activation and annexin V positive staining. In vitro and in vivo inhibition of caspase 8 significantly mitigated AM cell death, implicating apoptosis. In the extrinsic pathway of apoptosis, death receptors like members of the TNF receptor superfamily (TNFRSF) lead to signal transduction and caspase activation. Multiple TNFRSF transcripts were upregulated in AMs from infected lungs, including TNFRSF14 most strongly. TNFRSF14 (also known as HVEM) together with lymphotoxin β receptor (LTβR, also known as TNFRSF3) are receptors for TNF superfamily member 14 (TNFSF14, also known as LIGHT), leading to investigation of a TNFSF14-driven signaling axis in AM death.

During influenza infection, most of the apoptotic AMs stained positive for TNFRSF14 and/or LTβR. TNFRSF14 deficiency did not affect AM numbers, but LTβR deficiency prevented AM loss during influenza infection. The LTβR ligand TNFSF14 was increased in the bronchoalveolar lavage (BAL) fluid and was present throughout the lung parenchyma in influenza-infected mice, and patients with influenza or COVID-19 acute respiratory distress syndrome (ARDS) had elevated levels of TNFSF14 in BAL fluids compared with healthy controls. Recombinant TNFSF14 induced caspase activation and apoptosis in both mouse and human AMs. Deletion or blockade of TNFSF14 in mice decreased caspase activation and led to maintenance of the AM population during influenza infection. Thus, TNFSF14 was sufficient for AM death, and both TNFSF14 and LTβR were necessary for the AM death observed during influenza infection.

Single-cell RNA-Seq suggested neutrophils as sources of TNFSF14 in the infected mouse lung, and airspace neutrophils from patients with influenza ARDS had increased TNFSF14 expression. Depletion of neutrophils reduced soluble TNFSF14 and rescued the AM population in influenza-infected mice, implicating neutrophil-derived TNFSF14 as triggering AM cell death.

After identifying the TNFSF14/ LTβR pathway as mediating AM death during influenza infection, the authors asked whether the pathway contributes to bacterial superinfection severity. During secondary pneumococcal infection of the influenza-infected lung, TNFSF14-deficient mice displayed significantly increased AM abundance, decreased bacterial burdens, mitigated weight loss, and improved survival. Neutralization with anti-TNFSF14 antibodies before bacterial infection was similarly protective. Most impressively, the adoptive transfer of LTβR-deficient AMs during bacterial superinfection improved the survival of superinfected mice, while the adoptive transfers of WT or TNFRS14-deficient AMs did not. Thus, AMs that could not receive LTβR signaling were protective during superinfection.

**Figure 1:**
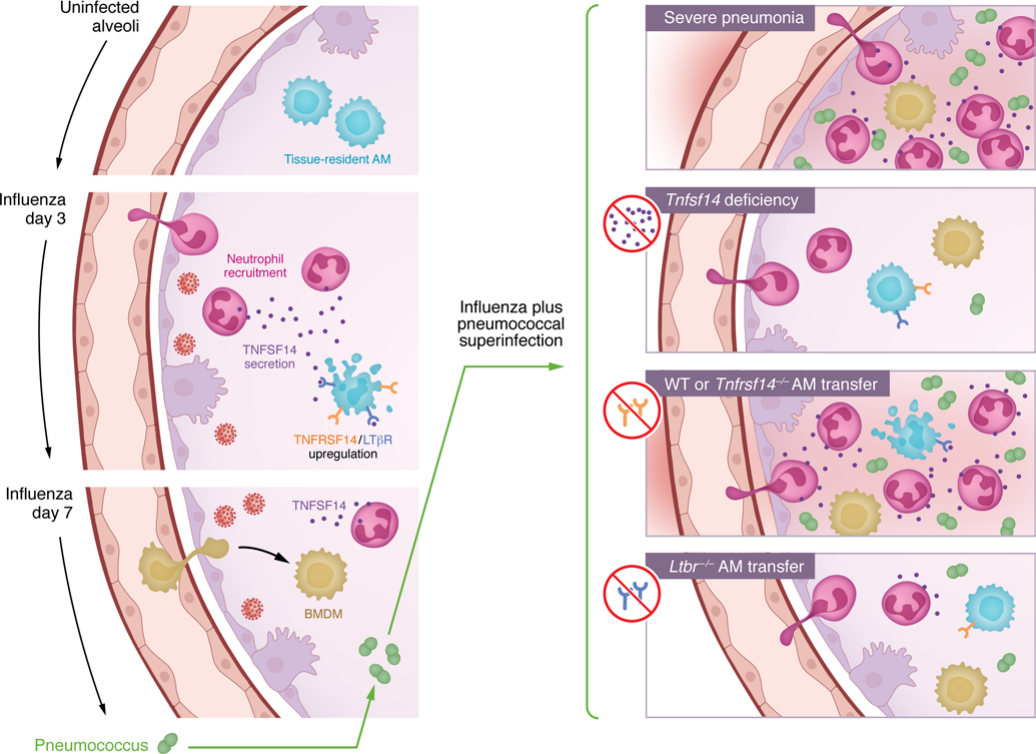
During influenza pneumonia, neutrophil-derived TNFSF14 uses the LTβR to trigger AM apoptosis, which increases susceptibility to severe bacterial superinfection. Studies by Malainou et. al. showed that neutrophils in the influenza-infected lung produced TNFSF14, whereas AMs increased expression of the TNFSF14 receptors TNFRSF14 and LTβR. TNFSF14 stimulated AM apoptosis primarily through the LTβR (8). These AM-depleted, influenza-infected lungs were susceptible to severe pneumococcal superinfection. However, deficiency of TNFSF14 during influenza infection (or its blockade prior to pneumococcal infection) made bacterial superinfection less severe. Although the adoptive transfer of WT or TNFRSF14-deficient AMs did not alter superinfection severity, the adoptive transfer of AMs lacking the LTβR (hence resistant to TNSF14-induced apoptosis) was sufficient to minimize the severity of superinfection. BMDM, bone marrow–derived macrophage.

Altogether, the data from Malainou et al. (8) compellingly reveal a mechanism shared by mice and humans in which neutrophil-derived TNFSF14 signals through the LTβR on AMs to trigger cell death during influenza infection, compromising host defense against secondary pneumococcal infection (Figure 1). The observations that interrupting TNFSF14 or delivering LTβR-deficient AMs can be lifesaving during pneumococcal superinfection of influenza-infected mice suggest that AMs do something in these superinfected lungs that is profoundly helpful.

## Implications and arising questions

While these data (8) demonstrate that the loss of AMs in influenza-infected lungs makes pneumococcal superinfection more severe, the AM roles responsible for protection against superinfection are uncertain. The ability of AMs to ingest and kill bacteria is plausibly an important role that is missing after TNFSF14-induced apoptosis, but many other phagocytes are also present in these influenza-infected lungs. Other activities of AMs, like their orchestration of the local immune response and of local resolution and repair (1, 2), may be as important or more important for the outcomes of bacterial superinfection. Unique required roles for AMs in curbing superinfection remain to be identified.

AM death is characteristic of pneumonias in which it has been studied and can occur via multiple mechanisms. For example, during pneumococcal pneumonia, AM apoptosis can be induced by high numbers of bacteria (9) or, in a manner similar to the findings by Malainou et al. (8), by neutrophil-derived TNFSF10 (also known as TRAIL) binding to TNFRSF10B (also known as DR5) on AMs (10). Many different viruses predispose to many types of superinfections. Circumventing AM death may depend on knowing the pathways responsible in a specific infectious setting. TNFSF14 associates with viral infections other than influenza (11, 12), so interrupting TNFSF14/LTβR signaling may have broader applicability for preventing bacterial superinfections secondary to respiratory viruses.

Blocking TNFSF14 or the LTβR during severe viral pneumonia is intriguing as a potential therapeutic approach. In a phase II clinical trial that tested the effects of a TNFSF14-blocking antibody in 62 patients with mild-to-moderate ARDS due to COVID-19 (13), the group of patients treated with anti-TNFSF14 had significantly decreased respiratory failure and mortality. However, whether any respiratory failure or mortality in this study may have involved bacterial superinfection was not reported. TNFSF14 or LTβR blockade might also have benefits for post-acute sequelae. For mice exposed to chronic cigarette smoke, a soluble LTβR protein that inhibits TNFSF14-LTβR interaction ameliorates tertiary lymphoid structures, damage-associated transitional epithelial cells, fibrosis, and emphysema (14), all of which can be post-acute sequelae of pneumonia. Cytokines from chronically activated CD8^+^ T cells are responsible for stimulating the monocyte-derived macrophages and aberrant epithelial cells that drive lung fibrosis after viral pneumonia (15, 16), so the roles of TNFSF14 in limiting contraction of activated CD8^+^ T lymphocytes and enhancing their lung residency (17) might mean that these adverse pulmonary sequelae would be mitigated by TNFSF14 blockade during infection. Along with evidence of roles for TNFSF14 and LTβR in safeguarding AMs (8), there is enthusiasm for further considering TNFSF14 or LTβR blockade in viral pneumonias.

Skepticism about potential therapeutic approaches targeting TNFSF14 or the LTβR is also warranted. In studies of mice that recovered from prior infections, which model the lung-resident immunity that helps protect the lungs of healthy young adult humans, AMs display very different phenotypes compared with the AMs of naive mice (3–7), and infection-experienced mice can be less reliant on AMs for eliminating pneumococci compared with infection-naive mice (4); thus, the roles of AMs in protecting naive mice against superinfection (8) may not directly translate to more experienced lungs, a context more relevant to most humans. Also, immune functions that may be compromised by acute inhibition of TNFSF14 or the LTβR bear consideration. In humans, biallelic loss-of-function LTβR mutations cause a primary immunodeficiency including hypogammaglobulinemia with diminished memory B cells, Tregs, and follicular Th cells (18). TNFSF14 helps program CD8^+^ T cells into memory and lung-resident states (17), which are pivotal to immune defense in healthy young adults (19). Therefore, LTβR or TNFSF14 blockade during a respiratory infection may compromise future adaptive immune defenses against related respiratory pathogens. Additionally, apoptosis of AMs is a method of killing microbes and is essential for effective defense against some infections, including pneumococcal infections (9, 10). The beneficial functions of AM apoptosis may be lost with TNFSF14 or LTβR blockade.

## Summary and outlook

Malainou et al. identified neutrophil-derived TNFSF14 signaling through LTβR on AMs as a trigger for AM apoptosis that was responsible for AM loss during influenza pneumonia, and they further demonstrated that interrupting TNFSF14/ LTβR signaling can protect mice during pneumococcal superinfection. Analyses of patient samples and cultured human AMs support the translational relevance of these findings. Further studies may help determine whether and when TNFSF14 or LTβR blockade might have clinical utility for respiratory viral infections.

## Funding support

This work is the result of NIH funding, in whole or in part, and is subject to the NIH Public Access Policy. Through acceptance of this federal funding, the NIH has been given a right to make the work publicly available in PubMed Central.

- NIH F31 HL178234 (to EMRA).
- NIH R01 HL171499 and R01 AI162850 (to JPM).

## Version 1 01/16/2026

Electronic publication
